# Comparison of 2 Triage Scoring Guidelines for Allocation of Mechanical Ventilators

**DOI:** 10.1001/jamanetworkopen.2020.29250

**Published:** 2020-12-14

**Authors:** Hannah Wunsch, Andrea D. Hill, Nicholas Bosch, Neill K. J. Adhikari, Gordon Rubenfeld, Allan Walkey, Bruno L. Ferreyro, Bourke W. Tillmann, Andre C. K. B. Amaral, Damon C. Scales, Eddy Fan, Brian H. Cuthbertson, Robert A. Fowler

**Affiliations:** 1 Department of Critical Care Medicine, Sunnybrook Health Sciences Centre, Toronto, Ontario, Canada; 2 Sunnybrook Research Institute, Toronto, Ontario, Canada; 3 ICES, Toronto, Ontario, Canada; 4 Interdepartmental Division of Critical Care Medicine, University of Toronto, Toronto, Ontario, Canada; 5 Department of Anesthesia, University of Toronto, Toronto, Ontario, Canada; 6 Department of Anesthesiology, Columbia University, New York, New York; 7 Department of Medicine, Boston University School of Medicine, Boston, Massachusetts; 8 Department of Medicine, University of Toronto, Toronto, Ontario, Canada; 9 University Health Network and Sinai Health System, Toronto, Ontario, Canada; 10 Li Ka Shing Knowledge Institute of St Michael’s Hospital, Toronto, Ontario, Canada

## Abstract

**Importance:**

In the current setting of the coronavirus disease 2019 pandemic, there is concern for the possible need for triage criteria for ventilator allocation; to our knowledge, the implications of using specific criteria have never been assessed.

**Objective:**

To determine which and how many admissions to intensive care units are identified as having the lowest priority for ventilator allocation using 2 distinct sets of proposed triage criteria.

**Design, Setting, and Participants:**

This retrospective cohort study conducted in spring 2020 used data collected from US hospitals and reported in the Philips eICU Collaborative Research Database. Adult admissions (N = 40 439) to 291 intensive care units from 2014 to 2015 who received mechanical ventilation and were not elective surgery patients were included.

**Exposures:**

New York State triage criteria and original triage criteria proposed by White and Lo.

**Main Outcomes and Measures:**

Sequential Organ Failure Assessment (SOFA) scores were calculated for admissions. The proportion of patients who met initial criteria for the lowest level of priority for mechanical ventilation using each set of criteria and their characteristics and outcomes were assessed. Agreement was compared between the 2 sets of triage criteria, recognizing differences in stated criteria aims.

**Results:**

Among 40 439 intensive care unit admissions of patients who received mechanical ventilation, the mean (SD) age was 62.6 (16.6) years, 54.9% were male, and the mean (SD) SOFA score was 4.5 (3.7). Using the New York State triage criteria, 8.9% (95% CI, 8.7%-9.2%) were in the lowest priority category; these lowest priority admissions had a mean (SD) age of 62.9 (16.6) years, used a median (interquartile range) of 57.3 (20.1-133.5) ventilator hours each, and had a hospital survival rate of 38.6% (95% CI, 37.0%-40.2%). Using the White and Lo triage criteria, 4.3% (95% CI, 4.1%-4.5%) were in the lowest priority category; these admissions had a mean (SD) age of 68.6 (13.2) years, used a median (interquartile range) of 61.7 (24.3-142.8) ventilator hours each, and had a hospital survival rate of 56.2% (95% CI, 53.8%-58.7%). Only 655 admissions (1.6%) were in the lowest priority category for both guidelines, with the κ statistic for agreement equal to 0.20 (95% CI, 0.18-0.21).

**Conclusions and Relevance:**

Use of 2 initially proposed ventilator triage guidelines identified approximately 1 in every 10 to 25 admissions as having the lowest priority for ventilator allocation, with little agreement. Clinical assessment of different potential criteria for triage decisions in critically ill populations is important to ensure valid and equitable allocation of resources.

## Key Points

**Question:**

What are the characteristics of intensive care unit admissions identified by 2 proposed pandemic ventilator allocation triage guidelines using Sequential Organ Failure Assessment scores when applied retrospectively to critically ill US patients who received mechanical ventilation?

**Findings:**

In this cohort study of 40 439 admissions to intensive care units that received mechanical ventilation, the New York State guideline identified 9% who would likely meet criteria for the lowest priority for ventilator allocation compared with 4% from the original White and Lo guideline. Only 655 admissions (1.6%) were in the lowest priority category for both guidelines, with 39% survival to hospital discharge for admissions identified as lowest priority using the New York State guideline compared with 56% for admissions identified using White and Lo.

**Meaning:**

Two distinct approaches to triage for mechanical ventilation showed little agreement, suggesting that further clinical assessment of different potential criteria for triage decisions is important to ensure equitable allocation of resources.

## Teaser

This cohort study compares the New York State Ventilator Allocation Guideline with the original triage criteria proposed by White and Lo to determine which and how many admissions to US intensive care units are identified as having the lowest priority for ventilator allocation.

## Introduction

The potential need to ration mechanical ventilators has been a worldwide focus during the coronavirus disease 2019 (COVID-19) pandemic.^1,2^ Many regions and countries have been forced to address the possible need for ventilator allocation, which refers to identification of critically ill patients for whom mechanical ventilation should be withheld or withdrawn if it has already been initiated.^3^ Different variations of triage criteria for this rationing have been proposed. In the US, 2 of the most prominent triage criteria are the New York State protocol^4^ and one initially proposed by White and Lo.^5^ Both protocols require assessment of a combination of burden of comorbidities and severity of illness on admission.

Despite extensive literature on resource allocation in pandemics, these criteria have not been tested on a large scale in high-income countries. It is unclear how many individuals would meet different triage criteria, which of the specific criteria would most commonly be met, and how many ventilators would be made available if these criteria were applied to deny care to patients deemed less likely to benefit. Therefore, we sought to apply these criteria to a heterogeneous cohort of patients across US intensive care units (ICUs) before the current COVID-19 pandemic as a first step to understanding the impact of applying these criteria to a critically ill population. Specifically, we assessed all admissions to ICUs for patients who received mechanical ventilation to determine how many would meet initial criteria for withholding of mechanical ventilation depending on the level of triage activated and then how many would meet criteria at a later time for withdrawal of mechanical ventilation.

## Methods

### Overview

We assessed triage criteria from 2 documents. The first is the New York State Ventilator Allocation Guidelines.^4^ The second is the original guideline proposed by White and Lo (referred to herein as the White and Lo guideline).^5^ This second guideline was subsequently updated on April 22, 2020, owing to concerns raised that it violated US antidiscrimination laws. However, the new version cannot be easily operationalized, and other guidelines based on it (eg, Colorado^6^) have fallen back on use of specific diagnoses to make it feasible to apply it to individual patients. Therefore, for this study, we have chosen to assess the original guideline. For each, we extracted explicit criteria used to determine patient assignment to each level of priority for ventilator allocation. We then applied these criteria to a cohort of ICU admissions from a non–COVID-19 period. This study adheres to the Strengthening the Reporting of Observational Studies in Epidemiology (STROBE) reporting guideline for cohort studies. This study used deidentified and publicly available data.^7^ Ethics approval was not applicable because the Philips eICU Collaborative Research Database^7^ is released under the Health Insurance Portability and Accountability Act safe harbor provision. The requirement to obtain informed patient consent was not applicable because the re-identification risk was certified as meeting safe harbor standards by Privacert (in Cambridge, Massachusetts).

### Cohort

This was a retrospective cohort study using the Philips eICU database,^7^ a large database of a random sample of ICU admissions to 291 ICUs in 208 US hospitals participating in Philips’ telehealth program from 2014 to 2015. We identified adult admissions (aged ≥18 years) for patients who received mechanical ventilation at any time during their ICU stay. We excluded admissions of patients who were never ventilated, those admitted after elective surgery, and anyone who had inaccurate data for included variables (eFigure 1 in the Supplement). Because patients could be admitted to the ICU more than once during a hospitalization and would on each admission be eligible for assessment regarding triage level, we included all admissions to the ICU.

### Outcomes

The outcomes assessed included (1) proportion of all admissions mechanically ventilated and patients who met each level of priority, (2) distribution of specific criteria that patients met for each level of priority, (3) proportion of all ventilator hours, (4) ICU mortality, and (5) hospital mortality. Among those who were not the lowest priority level on ICU admission, we also assessed how many additional patients met each level of priority 48 hours and 120 hours after admission. For outcomes and descriptors that could only occur once for a patient during a hospitalization (ie, hospital length of stay, hospital mortality, and discharge destination after the hospitalization), we restricted the analysis to the first ICU admission for each patient.

### New York State Guidelines

The New York State guidelines include 2 steps that are applied to patients on admission. The first is a list of exclusion criteria for adult patients with “medical conditions that result in immediate or near-immediate mortality even with aggressive therapy”^4^^(p57)^ (eTable 1 in the Supplement). To operationalize the exclusion criteria, we mapped them to available variables in the Philips eICU database (eTable 2 in the Supplement). The data available did not contain all of the granular criteria listed in the triage documents, and some criteria were not quantitative and required clinical interpretation to operationalize them using retrospective data (eTable 2 in the Supplement). The second step in the New York State guidelines is the calculation of a Sequential Organ Failure Assessment (SOFA) score on admission,^8^ followed by reassessment at 48 hours and 120 hours to detect any new onset of the stated medical conditions that warrant exclusion and to calculate the change in SOFA score (eTables 3 and 4 in the Supplement).

For the primary analysis, the SOFA score was calculated from the first available variables during the first 24 hours after admission to the ICU (SOFA first 24). The respiratory system score in the SOFA assessment includes points for mechanical ventilation. Because no details are given in the New York State guidelines regarding how to incorporate mechanical ventilation into the initial assessment when mechanical ventilation has been initiated before assessment, we calculated the SOFA score with the regular inclusion of points for mechanical ventilation. Patients with a SOFA score of exactly 7 were not categorized by the guidelines as published (eTable 3 in the Supplement); thus, we assigned these patients to the highest priority group. Missing data were assumed to be normal and not imputed, as is common in assessments of SOFA score^8^ and consistent with practice that would occur in real time regarding available data on a patient (eTable 5 in the Supplement). As a sensitivity analysis, we also recalculated the SOFA scores using the most extreme value for each variable in the first 24 hours after ICU admission (SOFA worst 24) and compared the classification of individuals by using the SOFA first 24 vs the SOFA worst 24.

### White and Lo Guidelines

The original White and Lo guidelines included a similar approach of assessing comorbidities and SOFA score. There were 2 different categorizations of major comorbid conditions and severe life-limiting conditions (eTable 6 in the Supplement). We similarly mapped these conditions using available variables in the Philips eICU database (eTable 7 in the Supplement). However, this approach did not create exclusions for specific conditions; instead, a certain number of points were allocated for each condition and added to points from the SOFA score (eTables 8 and 9 in the Supplement). Although reassessment is recommended, no specific times for reassessment or explicit criteria for withdrawal of therapies are given; thus, we did not apply any reassessments with the White and Lo guidelines.

### Statistical Analysis

We first summarized the characteristics of the ICU admission cohort, including age, sex, race/ethnicity (White, Black, Asian, Hispanic, or unknown), Acute Physiology and Chronic Health Evaluation (APACHE) IV measure of severity of illness,^9^ and SOFA score, as well as care preferences prior to or within the first 24 hours after admission to the ICU (defined as full therapy, do not resuscitate, or other [further] limitations on life support or other care, such as “no blood products” or “no vasopressors or inotropes”). We used proportions for categorical data and mean (SD) values or median values (interquartile ranges) for continuous data, as appropriate. Next, we calculated individual exclusions and comorbidities. Some admissions met more than 1 exclusion criterion or had more than 1 major comorbidity. We calculated the SOFA scores for all admissions using the SOFA first 24 as the primary assessment and the SOFA worst 24 as a secondary assessment and applied the triage criteria of each guideline to assign each admission to a level of priority for receipt of mechanical ventilation (lowest, intermediate, and highest). We then assessed the characteristics, resource use, and outcomes of the group of admissions in each priority level. We calculated the proportion of total ventilator hours for all patients in the ICU that were taken up by the lowest priority group to understand how many ventilator hours would be potentially “freed up” by not offering mechanical ventilation to this group. We compared differences between the lowest and highest priority groups using standardized differences, with a difference of less than 0.1 considered a negligible imbalance.^10^ Because we included all admissions (including multiple admissions by the same individual during the same hospitalization), we also performed a sensitivity analysis comparing agreement between the 2 sets of criteria but restricting the analysis to only the first ICU admission during the hospitalization.

#### Reassessment of Patients at 48 and 120 Hours

We applied the rules for reassessment of admissions at 48 and 120 hours using the New York State guidelines to determine the classification of admissions at these times for patients remaining in the ICU. We did not have an accurate way to determine new diagnoses or other changes in status that would allow someone to newly meet the exclusion criteria; thus, we did not apply this portion of the reassessment.

#### Comparison of Guidelines

We sought to determine whether these 2 different sets of triage criteria would identify the same or different admissions for allocation to the lowest priority group. We assessed the chance-corrected agreement between triage guidelines for assignment to the lowest priority category for mechanical ventilation using the κ statistic. All analyses were performed using SAS Enterprise Guide, version 7.1 (SAS Institute Inc), pgAdmin 4, version 3.2 (pgAdmin Development Team), and R studio, version 1.1.456 (R Project for Statistical Computing). The study was conducted in spring 2020.

## Results

### Cohort

After exclusions, the cohort consisted of 40 439 admissions to the ICU for patients who received mechanical ventilation during their ICU stay (eFigure 1 in the Supplement); the mean (SD) age was 62.6 (16.6) years, 54.9% were male, and three-quarters (75.9%) were White individuals (Table 1). The mean (SD) APACHE IV predicted mortality was 27.9% (26.1%). Mechanical ventilation was initiated in 33.8% of admissions before the day of ICU admission and 66.2% on or after ICU admission, with a median (interquartile range) duration of mechanical ventilation of 46.7 (17.0-122.9) hours. In the first 24 hours in the ICU, 87.9% of admissions were identified as receiving full therapy, 7.9% had a do-not-resuscitate order, and 4.2% had some other limitation on life support or other care. Overall hospital mortality (n = 8795) was 23.5%.

**Table 1.** Characteristics of Patients Who Received Invasive Mechanical Ventilation at Any Time During the Hospital Stay^a^

Characteristic	Total cohort, No. (%)
No.	40 439
Age, mean (SD), y^b^	62.6 (16.6)
Male sex	22 191 (54.9)
Race/ethnicity	
White	30 337 (75.9)
Black	5099 (12.8)
Asian	561 (1.4)
Hispanic	1456 (3.6)
Other/unknown	2514 (6.3)
ICU type	
Medical	4697 (11.6)
Medical/surgical	22 265 (55.1)
Surgical	2789 (6.9)
Neurological	3064 (7.6)
Cardiac/cardiothoracic	7624 (18.9)
APACHE IV score, mean (SD)	73.1 (30.7)
APACHE IV predicted mortality, mean (SD), %	27.9 (26.1)
Sequential Organ Failure Assessment score	
First value in first 24 h of ICU admission	
Mean (SD)	4.5 (3.7)
Median (IQR)	4 (1-7)
Worst value in first 24 h of ICU admission	
Mean (SD)	5.5 (3.9)
Median (IQR)	5 (2-8)
Care preferences prior to and within 24 h of ICU admission	
Full therapy	35 552 (87.9)
Do not resuscitate	3201 (7.9)
Limitations on life support or other care	1686 (4.2)
Invasive mechanical ventilation before ICU admission	13 666 (33.8)
Time from initiation of invasive mechanical ventilation to ICU admission, median (IQR), min	106 (21-222)
Invasive mechanical ventilation on or after ICU admission	26 773 (66.2)
Time from ICU admission to initiation of invasive mechanical ventilation, median (IQR), min	110 (21-871)
Total duration of ventilation, median (IQR), h	46.7 (17.0-122.9)
ICU length of stay, median (IQR), d	3.3 (1.7-6.7)
ICU mortality	6723 (16.6)
Hospitalization outcome^c^	
Hospital length of stay, median (IQR), d	7.7 (3.9-14.0)
Hospital mortality	8795 (23.5)
Discharge disposition among hospital survivors	
Home	14 123 (49.5)
Other hospital	2051 (7.2)
Rehabilitation	2253 (7.9)
Skilled nursing facility	6171 (21.6)
Other	3942 (13.8)

Abbreviations: APACHE, Acute Physiology and Chronic Health Evaluation; ICU, intensive care unit; IQR, interquartile range.

^a^ For missing data information, see eTable 5 in the Supplement.

^b^ Patients aged older than 89 years set to 90 years per requirements for public data access.

^c^ Restricted to the first ICU stay that included invasive mechanical ventilation during the hospitalization (n = 37 736).

### Relevant Comorbidities

As applied to this cohort, 5.4% (95% CI, 5.2%-5.6%) of admissions met at least 1 exclusion criterion in the New York State triage guidelines (Table 2). The most frequent reason was refractory or unwitnessed cardiac arrest (3.9%; 95% CI, 3.7%-4.1%), with less than 1% meeting each of the other exclusion criteria. Using the White and Lo criteria, 17.7% (95% CI, 17.3%-18.1%) had at least 1 of the major comorbidities, and 8.7% (95% CI, 8.4%-9.0%) had a severe life-limiting comorbidity that contributed points to the triage priority score. The most common comorbidities were chronic lung disease and dementia (Table 2).

**Table 2.** Patients With Each Criterion for Either Exclusion (New York State Guidelines) or Receipt of Comorbidity Points (White and Lo Guidelines)

Clinical condition	Met criterion, % (n = 40 439)		
	No	Yes	95% CI
Exclusion criteria (New York State)			
Any	94.6	5.4	5.2-5.6
Cardiac arrest	96.1	3.9	3.7-4.1
Irreversible hypotension	99.3	0.7	0.6-0.7
Traumatic brain injury with no motor response	99.3	0.7	0.6-0.8
Severe burns^a^			
Other conditions	99.3	0.7	0.7-0.8
Major comorbidities (White and Lo)			
Any	82.3	17.7	17.3-18.1
Moderate Alzheimer or related dementia	98.1	1.9	1.8-2.0
Malignant neoplasm with <10 y expected survival	98.7	1.3	1.2-1.4
NYHA class III heart failure	99.7	0.3	0.3-0.4
Moderate to severe chronic lung disease	90.5	9.5	9.2-9.8
ESKD and aged <75 y	97.4	2.6	2.5-2.8
Severe multivessel CAD	98.8	1.2	1.1-1.3
Cirrhosis with history of decompensation	97.9	2.1	2.0-2.3
Severe life-limiting comorbidities (White and Lo)			
Any	91.3	8.7	8.4-9.0
Severe Alzheimer or related dementia	98.4	1.6	1.4-1.7
Cancer with only palliative interventions	99.6	0.4	0.4-0.5
NYHA class IV heart failure + frailty	99.6	0.4	0.4-0.5
Severe chronic lung disease and evidence of frailty	94.3	5.7	5.4-5.9
ESKD and aged >75 y^b^	99.3	0.7	0.6-0.8
Cirrhosis with MELD ≥20, ineligible for transplant	99.9	0.1	0.1-0.2

Abbreviations: CAD, coronary artery disease; ESKD, end-stage kidney disease; MELD, Model for End-Stage Liver Disease; NYHA, New York Heart Association.

^a^ No patient met this criterion.

^b^ Because 27 individuals aged 75 years were unspecified for classification in either ESKD category, we included them in the severe life-limiting comorbidities category.

### SOFA Scores

The mean (SD) SOFA first 24 score for the cohort was 4.5 (3.7), with a median of 4 and a range from 0 to 21 (eFigure 2 in the Supplement). When recalculated using the worst value in the first 24 hours after ICU admission, the mean (SD) was 5.5 (3.9), with a median of 5 and a range from 0 to 22. Sixty percent of the cohort changed to a different SOFA score when using SOFA worst 24 rather than SOFA first 24, with 18.6% changing by 2 or more points.

### Classification of Priority of Admissions

Using the New York State guidelines, 8.9% (95% CI, 8.7%-9.2%) of admissions would be classified in the lowest priority category for mechanical ventilation and 77.1% (95% CI, 76.7%-77.5%) in the highest priority category (Table 3). The White and Lo guidelines identified 4.3% (95% CI, 4.1%-4.5%) of the admissions as meeting criteria for the lowest priority category for mechanical ventilation and 81.3% (95% CI, 80.9%-81.7%) as highest priority. Reclassification of admissions using the SOFA worst 24 meant that 11.3% (95% CI, 11.0%-11.6%) of admissions met criteria using the New York State guidelines and 5.7% (95% CI, 5.5%-5.9%) using the White and Lo guidelines (eTable 10 in the Supplement). For admitted patients still in the ICU after 48 hours and not already identified as low priority (n = 25 383) using the New York State guidelines, an additional 1193 admissions (4.7% [95% CI, 4.4%-5.0%] of those remaining) would have been reclassified as lowest priority; by 120 hours (n = 6572), a further 1862 (28.3% [95% CI, 27.2%-29.4%] of those remaining) would have been reclassified as lowest priority (Figure).

**Table 3.** Characteristics of Patients by Categorization for Mechanical Ventilation Using Each Triage System

Characteristic^a^	Categorization of priority for mechanical ventilation within first 24 h of ICU admission, No. (%)							
	New York State				White and Lo			
	Lowest	Intermediate	Highest	Standardized difference^b^	Lowest	Intermediate	Highest	Standardized difference^b^
No. of patients, (%) [95% CI]	3612 (8.9) [8.7-9.2]	5640 (14.0) [13.6-14.3]	31 187 (77.1) [76.7-77.5]		1738 (4.3) [4.1-4.5]	5831 (14.4) [14.1-14.8]	32 870 (81.3) [80.9-81.7]	
Proportion of total ventilation hours, % (95% CI)	10.5 (10.3-10.6)	18.6 (18.4-18.8)	71.0 (70.7-71.2)		5.0 (4.9-5.1)	16.5 (16.3-16.7)	78.5 (78.3-78.7)	
Age, mean (SD), y	62.9 (16.6)	63.4 (15.5)	62.4 (16.8)	0.03	68.6 (13.2)	65.5 (13.7)	61.8 (17.1)	0.44
Male sex	2148 (59.6)	3330 (59.1)	16 713 (53.6)	0.12	964 (55.5)	3079 (52.8)	18 148 (55.2)	0.01
Race/ethnicity								
White	2618 (73.2)	4167 (74.6)	23 552 (76.5)	0.13	1244 (71.9)	4402 (76.2)	24 691 (76.1)	0.16
Black	444 (12.4)	709 (12.7)	3946 (12.8)		242 (14.0)	754 (13.1)	4103 (12.6)	
Asian	48 (1.3)	54 (1.0)	459 (1.5)		13 (0.8)	70 (1.2)	478 (1.5)	
Hispanic	187 (5.2)	256 (4.6)	1013 (3.3)		110 (6.4)	209 (3.6)	1137 (3.5)	
Other or unknown	280 (7.8)	403 (7.2)	1831 (5.9)		120 (6.9)	340 (5.9)	2054 (6.3)	
ICU type								
Medical	357 (9.9)	643 (11.4)	3697 (11.9)	0.22	191 (11.0)	770 (13.2)	3736 (11.4)	0.32
Medical/surgical	2042 (56.5)	3384 (60.0)	16 839 (54.0)		1106 (63.6)	3372 (57.8)	17 787 (54.1)	
Surgical	218 (6.0)	309 (5.5)	2262 (7.3)		66 (3.8)	331 (5.7)	2392 (7.3)	
Neurological	152 (4.2)	241 (4.3)	2671 (8.6)		44 (2.5)	227 (3.9)	2793 (8.5)	
Cardiac/cardiothoracic	843 (23.3)	1063 (18.8)	5718 (18.3)		331 (19.0)	1131 (19.4)	6162 (18.7)	
APACHE IV score, mean (SD)	120.4 (27.3)	91.7 (25.7)	63.7 (24.1)	2.20	101.2 (31.9)	87.0 (33.2)	68.9 (28.5)	1.07
APACHE IV predicted mortality, mean (SD), %	71.3 (22.9)	41.0 (23.9)	19.8 (19.4)	2.43	51.4 (27.6)	39.8 (28.7)	24.3 (24.0)	1.05
SOFA score, mean (SD)	10.1 (4.2)	9.1 (1.1)	3.1 (2.4)	2.06	9.6 (3.1)	7.0 (4.4)	3.8 (3.1)	1.88
Care preferences prior to and within 24 h of ICU admission								
Full therapy	2812 (77.9)	4907 (87.0)	27 833 (89.2)	0.31	1341 (77.2)	4827 (82.8)	29 384 (89.4)	0.33
Do not resuscitate	545 (15.1)	495 (8.8)	2161 (6.9)		253 (14.6)	650 (11.1)	2298 (7.0)	
Limitations on life support or other care	255 (7.1)	238 (4.2)	1193 (3.8)		144 (8.3)	354 (6.1)	1188 (3.6)	
Care preferences prior to and within 24 h of ICU discharge								
Full therapy	1915 (53.0)	3822 (67.8)	24 411 (78.3)	0.55	951 (54.7)	3790 (65.0)	25 407 (77.3)	0.49
Do not resuscitate	922 (25.5)	953 (16.9)	3630 (11.6)		394 (22.7)	1068 (18.3)	4043 (12.3)	
Limitations on life support or other care	775 (21.5)	865 (15.3)	3146 (10.1)		393 (22.6)	973 (16.7)	3420 (10.4)	
Duration of ventilation, median (IQR), h	57.3 (20.1-133.5)	67.3 (27.1-160.2)	42.1 (15.1-114.0)	0.19	61.7 (24.3-142.8)	56.5 (21.8-136.5)	44.6 (15.9-118.9)	0.21
Survived to ICU discharge	90.8 (36.8-196.0)	73.2 (30.9-166.7)	41.1 (15.2-110.2)	0.55	68.6 (29.5-152.1)	64.1 (25.2-145.4)	43.6 (16.0-117.1)	0.33
Died prior to ICU discharge	36.2 (14.7-80.9)	51.6 (18.8-135.2)	57.2 (14.8-148.2)	0.24	41.2 (18.0-113.4)	38.9 (15.6-104.0)	50.8 (15.1-129.8)	0.04
ICU length of stay, median (IQR), d	3.1 (1.1-7.0)	4.4 (2.2-8.3)	3.1 (1.7-6.3)	0.05	3.6 (1.9-7.1)	3.5 (1.7-7.1)	3.3 (1.7-6.6)	0.08
ICU mortality	1851 (51.3)	1388 (24.6)	3484 (11.2)	0.96	585 (33.7)	1432 (24.6)	4706 (14.3)	0.47
Hospitalization outcomes^c^								
Hospital length of stay, median (IQR), d	5.4 (1.8-13.2)	9.5 (4.8-16.3)	7.7 (4.0-13.7)	0.28	7.8 (3.6-13.8)	7.9 (3.9-14.2)	7.7 (3.9-13.9)	0.04
Hospital mortality	2120 (61.4)	1715 (33.2)	4960 (17.2)	1.01	709 (43.8)	1780 (33.0)	6306 (20.7)	0.51
Discharge disposition								
Home	468 (35.2)	1407 (40.9)	12 248 (51.5)	0.35	355 (39.0)	1554 (43.2)	12 214 (50.8)	0.29
Other hospital	144 (10.8)	314 (9.1)	1593 (6.7)		78 (8.6)	305 (8.5)	1668 (6.9)	
Rehabilitation	151 (11.4)	283 (8.2)	1819 (7.7)		55 (6.0)	248 (6.9)	1950 (8.1)	
Skilled nursing facility	325 (24.5)	901 (26.2)	4945 (20.8)		281 (30.9)	935 (26.0)	4955 (20.6)	
Other	241 (18.1)	535 (15.6)	3166 (13.3)		141 (15.5)	552 (15.4)	3249 (13.5)	

Abbreviations: APACHE, Acute Physiology and Chronic Health Evaluation; ICU, intensive care unit; IQR, interquartile range; SOFA, Sequential Organ Failure Assessment.

^a^ For missing data, see eTable 5 in the Supplement.

^b^ Comparison between lowest priority and highest priority categories; absolute differences higher than 0.1 are considered significant.

^c^ Restricted to the first ICU stay that included mechanical ventilation during the hospitalization (n = 37 736).

**Figure.**
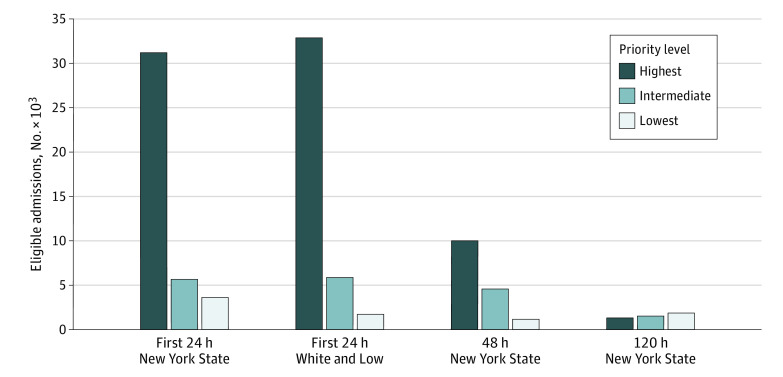
Number of Patients Meeting Each Ventilator Allocation Triage Level During the First 24 Hours of Admission to an Intensive Care Unit for the New York State and White and Lo Guidelines and for the New York State Guidelines 48 and 120 Hours After Admission

### Characteristics and Hospital Outcomes

The New York State guidelines identified a lowest priority group of admissions with a median (interquartile range) of 57.3 (20.1-133.5) ventilator hours each, representing 10.5% of ventilator hours of the total cohort. The lowest priority category identified by the White and Lo guidelines each used a median (interquartile range) of 61.7 (24.3-142.8) ventilator hours and a total of 5.0% of all ventilator hours of the cohort (Table 3). Using the New York State guidelines, those classified as lowest priority were of similar age as admitted patients assigned to other priority groups (Table 3) but with slightly more who were non-White and more with limitations on care. The APACHE IV predicted mortality was 71.3%. Actual hospital mortality (using only the first ICU admission) was 61.4% (hospital survival, 38.6%; 95% CI, 37.0%-40.2%); among those who survived, 35.2% were discharged home. This outcome was in contrast with the lowest priority group identified by the White and Lo guidelines, which was composed of slightly older admitted patients (mean [SD] age, 68.6 [13.2] years) and an increased percentage of non-White individuals but with a similar distribution of limitations on care in the first 24 hours after ICU admission. This group had lower predicted (51.4%) and actual (43.8%) mortality (hospital survival, 56.2%; 95% CI, 53.8%-58.7%). A similar proportion of survivors (39.0%) were discharged home.

### Agreement Between the 2 Triage Systems

There was poor agreement between the 2 triage systems regarding which admissions were identified as lowest priority for mechanical ventilation (κ = 0.20; 95% CI, 0.18-0.21), recognizing that the 2 proposed triage systems have unique aims in terms of values for identifying patients. Of the 3612 and 1738 admissions identified as lowest priority by the different scoring systems, only 655 met criteria in both (1.6% of the overall cohort) (Table 4). Using the New York State guidelines, 3.8% of all admissions identified as lowest priority would have been identified as intermediate priority and 3.6% as highest priority using White and Lo criteria. Similarly, 1.2% of admissions identified as lowest priority using White and Lo guidelines would have been identified as intermediate priority and 1.4% as highest priority using New York State guidelines. Characteristics of the admissions that met the lowest criteria using both criteria vs other patients who met criteria for lowest priority in each guideline for one set of criteria are given in eTable 11 in the Supplement. Restricting analyses to the first admission to the ICU during the hospitalization yielded the same agreement (1.6%; eTable 12 in the Supplement).

**Table 4.** Comparison of Agreement in Classification Regarding Admissions in Each Priority Category for Ventilation on Admission to the Intensive Care Unit^a^

New York State priority score	White and Lo priority score, No. (%)		
	6-8 (Lowest priority)	4-5 (Intermediate priority)	1-3 (Highest priority)
Lowest priority	655 (1.6)	1523 (3.8)	1434 (3.6)
Intermediate priority	503 (1.2)	1066 (2.6)	4071 (10.1)
Highest priority	580 (1.4)	3242 (8.0)	27 365 (67.7)

^a^ Percentages refer to the entire cohort. For agreement assessing the lowest priority vs other categories combined (intermediate priority and highest priority), κ = 0.20 (95% CI, 0.18-0.21).

## Discussion

When applied retrospectively to a cohort of critically ill patients without COVID-19, 2 different ventilator allocation guidelines identified 4.3% and 8.9% of admissions as meeting the lowest level of priority to receive mechanical ventilation. Patients who were assigned the lowest level of priority by these guidelines received 2 to 3 days of ventilation, and both guidelines identified a slightly higher percentage of non-White patients as lowest priority. However, the 2 triage criteria identified substantially different patients for initial consideration for withholding (or very early withdrawal) of mechanical ventilation.

Although many triage criteria exist, few studies have assessed the application of these criteria to actual patients. After the H1N1 pandemic in 2009, a few small studies assessed triage scores or tools for triage.^11,12,13^ However, those studies focused on predicting the need for mechanical ventilation rather than assessing which patients would be identified as lowest priority and determining their outcomes. In the present study, we identified challenges with attempting to apply these criteria—particularly SOFA scores—to patients retrospectively admitted to ICUs given that some life-sustaining interventions had already been applied. Our finding that the use of the worst value of a variable to calculate a SOFA score led to different scores for patients highlights the fluid nature of these scores and the challenges associated with hard cutoffs; a SOFA score may change markedly from before to after intubation in particular, with either deterioration or stabilization after intubation. Moreover, SOFA scores have been primarily validated for general intensive care or sepsis populations and not for isolated respiratory disease.^14,15^

The New York State criteria overall identified a sicker cohort. Because the overall goal of these triage criteria is to minimize allocation of a scarce resource to those who seem least likely to benefit, the New York State criteria appeared closer to achieving this goal. However, many admitted patients identified as lowest priority by both sets of criteria survived to at least hospital discharge. Moreover, we are unable to determine whether the choice to forgo mechanical ventilation in favor of treatments, such as prone positioning, may result in better outcomes for some patients, as has been raised as an area of discussion with regard to patients with COVID-19 and their treatment management.^16^ The triage criteria do explicitly state that they emphasize different priorities; the New York State criteria define survival by examining a patient’s short-term likelihood of surviving the acute medical episode and not by focusing on whether the patient may survive a given illness or disease in the long term (eg, years after the pandemic). By contrast, the White and Lo guidelines use an approach that is more focused on balancing short- and long-term risk of death.

Neither of these 2 guidelines included age as a criterion for allocation of resources, but guidelines such as those adopted by Colorado based on the White and Lo criteria do explicitly include age.^6^ There are differences of opinion regarding the appropriateness of allocation that may incorporate age in such decisions, and it is important to understand the consequences of different proposed criteria. Similarly, studies of patients with COVID-19 have highlighted a concerning pattern in the US, suggesting that minority groups, such as Black persons, may be disproportionately affected by COVID-19.^17^ An important aspect of triage criteria assessment is to determine whether the criteria may disadvantage certain groups. Although difficult to ascertain without application to patients with COVID-19 themselves, the New York State guidelines did not disproportionately identify Black patients, but both allocation schemes did, overall, identify more non-White patients in the lowest priority groups. Seemingly objective criteria for allocation will almost inevitably fail to address inequities (and likely perpetuate them) unless considerations of race/ethnicity and other relevant social factors are incorporated in the development and evaluation of the criteria.

### Limitations

This study has major limitations. We applied these criteria to a non–COVID-19 cohort of admissions who received mechanical ventilation. We recognize that the demographic characteristics and severity of illness (eg, SOFA scores) of patients admitted with COVID-19 may have a different distribution. In particular, patients with COVID-19 may represent a more homogeneous population that would allow for better agreement between different guidelines when applied and, owing to single organ failure, may more frequently have a low SOFA score. However, potential application of triage criteria would not be isolated to patients with COVID-19 but would be as relevant for patients represented in this cohort. Future work should assess more contemporary patients to determine what the application of these criteria would look like in a period with primarily COVID-19 admissions and how SOFA or other scores may need to be recalibrated for a COVID-19 population. We could not fully determine patients’ preferences for ventilation and other invasive support or the clinical criteria used for the decision to place a patient on a ventilator because we were limited by the use of retrospective data. We recognize that there can be substantial variability in these decisions that influence the cohort we assessed.^18^ We also had to approximate some of the criteria for comorbidities based on available data, and we had some missing data, particularly regarding APACHE IV, that we chose not to impute. We tried to be conservative in our approach to err on the side of undertriage rather than overtriage of individuals to the lowest priority categories. Of the many triage protocols proposed, we chose to assess only 2 that could be most easily operationalized using retrospective cohort data,^19^ and we examined only US criteria and patients. A preliminary triage proposal in Ontario, Canada, has a very different approach, which eschews the use of any severity of illness score in favor of a more detailed assessment of clinical condition and underlying diseases to determine who is at high risk of death.^20^ Such approaches may identify different patient populations. We also did not assess the actual number of ICU bed-days of these patients; given the large number of nonventilated patients in US ICUs as well as the complex reallocation of beds associated with reductions in elective surgery and other procedures, such calculation was outside the scope of this work.

Finally, we chose to focus on patients meeting the lowest priority criteria for mechanical ventilation. These guidelines are set up to assess different levels of priority, and in times of a true shortage, there may be escalation to withholding or withdrawal of mechanical ventilation in the intermediate category. Reassessment of patients would also occur. Because we did not have an accurate way to determine new diagnoses or other changes in status that would allow someone to newly meet the exclusion criteria, we did not apply this portion of the reassessment. Therefore, we have underestimated the number of patients who would have transitioned to the lowest priority category. We also chose to focus on the triage for mechanical ventilators. However, other equipment, such as dialysis machines, personal protective equipment, and personnel, may be the aspects of care that ultimately are in shortest supply.^21,22^

## Conclusions

This study assessed the application of 2 different ventilator triage criteria retrospectively to critically ill patients without COVID-19, showing the complexity of this application and the disparate choices that might be made depending on the triage criteria chosen. Allocation of life-saving resources in a pandemic is a challenging concept, and many competing interests would be involved in decisions to withhold or withdraw mechanical ventilation. We recognize that the criteria put forth in these guidelines were done so as suggestions rather than as hard-and-fast rules to be adopted across a cohort of patients by using administrative data. This study highlights the importance of assessing these approaches using actual patient data because it allows for determination of the ways that guidelines may be difficult to operationalize, whether certain groups may be disproportionately affected, and what further work is needed to refine these approaches for clinical use. In particular, the lack of agreement between the assessed guidelines highlights that these guidelines approach this allocation challenge in somewhat unique ways and lead to different choices.

## Notes

Supplement. **eFigure 1.** Flowchart of Exclusions for Cohort **eFigure 2.** Distribution of SOFA Scores Calculated Using the First Available Data in the 24 h After Admission to ICU vs Worst Values in the First 24 h for All Mechanically Ventilated Admissions **eTable 1.** List of Exclusion Criteria for Adult Patients for NY State Guidelines **eTable 2.** Operational Definitions for Exclusion Criteria for Adult Patients for NY State Guidelines, Using Data Available From Philips eICU Database **eTable 3.** Triage Level for Patients Based on Application of Criteria at Time of Admission to ICU Taken From NY State Guidelines **eTable 4.** Triage Level for Patients Based on Application of Criteria (A) at 48 h; (B) at 120 h Taken From NY State Guidelines **eTable 5.** Number and Percentage of Records Missing Data on Key Variables **eTable 6.** Original White & Lo Guidelines; Examples of Major Comorbidities and Severely Life Limiting Comorbidities **eTable 7.** Operational Definitions for Major and Severe Life-Limiting Diseases in White & Lo Guidelines, Using Data Available From Philips eICU Database **eTable 8.** Point Assignments for Original White & Lo Guidelines **eTable 9.** Triage Level for Patients Based on Application of Criteria at Time of Admission to ICU in Original White & Lo Guidelines **eTable 10.** Estimates of Percentage of Admissions Who Would Meet Lowest Priority Criteria for Mechanical Ventilation **eTable 11.** Characteristics of the Admissions Who Were Lowest Priority in Both Sets of Guidelines vs Other Patients Who Met Criteria for Lowest Priority in Each Guideline **eTable 12.** Comparison of Agreement in Classification Regarding Patients in Each Category for Priority for Ventilation on Admission to ICU—Restricted to the First ICU Admission During the Hospitalization **eReferences**

## References

[1] Emanuel EJ, Persad G, Upshur R, Fair allocation of scarce medical resources in the time of Covid-19. N Engl J Med. 2020;382(21):2049-2055. doi:10.1056/NEJMsb2005114 32202722

[2] Truog RD, Mitchell C, Daley GQ The toughest triage—allocating ventilators in a pandemic. N Engl J Med. 2020;382(21):1973-1975. doi:10.1056/NEJMp2005689 32202721

[3] Rosenbaum L Facing Covid-19 in Italy—ethics, logistics, and therapeutics on the epidemic’s front line. N Engl J Med. 2020;382(20):1873-1875. doi:10.1056/NEJMp2005492 32187459

[4] New York State Task Force on Life and the Law. Ventilator allocation guidelines. New York State Department of Health. Published November 2015. Accessed October 30, 2020. https://www.health.ny.gov/regulations/task_force/reports_publications/docs/ventilator_guidelines.pdf

[5] White DB, Lo B A framework for rationing ventilators and critical care beds during the COVID-19 pandemic. JAMA. 2020;323(18):1773-1774. doi:10.1001/jama.2020.5046 32219367

[6] Colorado Department of Public Health and Environment. Colorado crisis standards of care. Published 2019. Accessed May 10, 2020. https://www.colorado.gov/pacific/cdphe/colorado-crisis-standards-care

[7] Pollard TJ, Johnson AEW, Raffa JD, Celi LA, Mark RG, Badawi O The eICU Collaborative Research Database, a freely available multi-center database for critical care research. Sci Data. 2018;5:180178. doi:10.1038/sdata.2018.178 30204154 PMC6132188

[8] Vincent JL, de Mendonça A, Cantraine F, ; Working group on "sepsis-related problems" of the European Society of Intensive Care Medicine Use of the SOFA score to assess the incidence of organ dysfunction/failure in intensive care units: results of a multicenter, prospective study. Crit Care Med. 1998;26(11):1793-1800. doi:10.1097/00003246-199811000-00016 9824069

[9] Zimmerman JE, Kramer AA, McNair DS, Malila FM Acute Physiology and Chronic Health Evaluation (APACHE) IV: hospital mortality assessment for today’s critically ill patients. Crit Care Med. 2006;34(5):1297-1310. doi:10.1097/01.CCM.0000215112.84523.F0 16540951

[10] Austin PC Using the standardized difference to compare the prevalence of a binary variable between two groups in observational research. Commun Stat Simul Comput. 2009;38(6):1228-1234. doi:10.1080/03610910902859574

[11] Adeniji KA, Cusack R The Simple Triage Scoring System (STSS) successfully predicts mortality and critical care resource utilization in H1N1 pandemic flu: a retrospective analysis. Crit Care. 2011;15(1):R39. doi:10.1186/cc10001 21269458 PMC3221968

[12] Khan Z, Hulme J, Sherwood N An assessment of the validity of SOFA score based triage in H1N1 critically ill patients during an influenza pandemic. Anaesthesia. 2009;64(12):1283-1288. doi:10.1111/j.1365-2044.2009.06135.x 19860754

[13] Morton B, Tang L, Gale R, Performance of influenza-specific triage tools in an H1N1-positive cohort: P/F ratio better predicts the need for mechanical ventilation and critical care admission. Br J Anaesth. 2015;114(6):927-933. doi:10.1093/bja/aev042 25829394

[14] Ferreira FL, Bota DP, Bross A, Mélot C, Vincent JL Serial evaluation of the SOFA score to predict outcome in critically ill patients. JAMA. 2001;286(14):1754-1758. doi:10.1001/jama.286.14.1754 11594901

[15] Raith EP, Udy AA, Bailey M, ; Australian and New Zealand Intensive Care Society (ANZICS) Centre for Outcomes and Resource Evaluation (CORE) Prognostic accuracy of the SOFA score, SIRS criteria, and qSOFA score for in-hospital mortality among adults with suspected infection admitted to the intensive care unit. JAMA. 2017;317(3):290-300. doi:10.1001/jama.2016.20328 28114553

[16] Coppo A, Bellani G, Winterton D, Feasibility and physiological effects of prone positioning in non-intubated patients with acute respiratory failure due to COVID-19 (PRON-COVID): a prospective cohort study. Lancet Respir Med. 2020;8(8):765-774. doi:10.1016/S2213-2600(20)30268-X 32569585 PMC7304954

[17] Yancy CW COVID-19 and African Americans. JAMA. 2020;323(19):1891-1892. doi:10.1001/jama.2020.6548 32293639

[18] Wunsch H Mechanical ventilation in COVID-19: interpreting the current epidemiology. Am J Respir Crit Care Med. 2020;202(1):1-4. doi:10.1164/rccm.202004-1385ED 32402207 PMC7328308

[19] Antommaria AHM, Gibb TS, McGuire AL, Ventilator triage policies during the COVID-19 pandemic at U.S. hospitals associated with members of the Association of Bioethics Program Directors. Ann Intern Med. 2020;173(3):188-194. doi:10.7326/M20-1738 32330224 PMC7207244

[20] Ontario Health. Clinical triage protocol for major surge in COVID pandemic. Published March 28, 2020. Accessed October 30, 2020. https://caep.ca/wp-content/uploads/2020/04/Clinical-Triage-Protocol-for-Major-Surge-in-COVID-Pandemic-March-28-202.pdf

[21] Goldfarb DS, Benstein JA, Zhdanova O, Impending shortages of kidney replacement therapy for COVID-19 patients. Clin J Am Soc Nephrol. 2020;15(6):880-882. doi:10.2215/CJN.05180420 32345750 PMC7274293

[22] Kumaraiah D, Yip N, Ivascu N, Hill L Innovative ICU physician care models: Covid-19 pandemic at New York-Presbyterian. *NEJM Catalyst: Innovations in Care Delivery* Published April 28, 2020. Accessed November 2, 2020. https://catalyst.nejm.org/doi/pdf/10.1056/CAT.20.0158

