# Clarification of Triage Scoring Criteria

**DOI:** 10.1001/jamanetworkopen.2021.2183

**Published:** 2021-02-08

In the Original Investigation, “Comparison of 2 Triage Scoring Guidelines for Allocation of Mechanical Ventilators,”^1^ Published on December 14, 2020, clarifications were made to the description of the second set of criteria evaluated. The second set included 2 criteria selected from a 4-component allocation framework developed using principles of saving lives and saving life-years, which are referred to as save lives/life-years criteria. The article^1^ and its Editorial^2^ have been corrected to reflect this clarification.
