# Supplementary material for: Comparison of 2 Triage Scoring Guidelines for Allocation of Mechanical Ventilators
Source: JAMA Netw Open. 2020 Dec 14;3(12):e2029250. doi: 10.1001/jamanetworkopen.2020.29250 (PMC7737087; doi:10.1001/jamanetworkopen.2020.29250)
Supplement: Supplement. — eFigure 1. Flowchart of Exclusions for Cohort eFigure 2. Distribution of SOFA Scores Calculated Using the First Available Data in the 24 h After Admission to ICU vs Worst Values in the First 24 h for All Mechanically Ventilated Admissions eTable 1. List of Exclusion Criteria for Adult Patients for NY State Guidelines eTable 2. Operational Definitions for Exclusion Criteria for Adult Patients for NY State Guidelines, Using Data Available From Philips eICU Database eTable 3. Triage Level for Patients Based on Application of Criteria at Time of Admission to ICU Taken From NY State Guidelines eTable 4. Triage Level for Patients Based on Application of Criteria (A) at 48 h; (B) at 120 h Taken From NY State Guidelines eTable 5. Number and Percentage of Records Missing Data on Key Variables eTable 6. Original White & Lo Guidelines; Examples of Major Comorbidities and Severely Life Limiting Comorbidities eTable 7. Operational Definitions for Major and Severe Life-Limiting Diseases in White & Lo Guidelines, Using Data Available From Philips eICU Database eTable 8. Point Assignments for Original White & Lo Guidelines eTable 9. Triage Level for Patients Based on Application of Criteria at Time of Admission to ICU in Original White & Lo Guidelines eTable 10. Estimates of Percentage of Admissions Who Would Meet Lowest Priority Criteria for Mechanical Ventilation eTable 11. Characteristics of the Admissions Who Were Lowest Priority in Both Sets of Guidelines vs Other Patients Who Met Criteria for Lowest Priority in Each Guideline eTable 12. Comparison of Agreement in Classification Regarding Patients in Each Category for Priority for Ventilation on Admission to ICU—Restricted to the First ICU Admission During the Hospitalization eReferences [file jamanetwopen-e2029250-s001.pdf]

Supplementary Online Content

Wunsch H, Hill AD, Bosch N, et al. Comparison of 2 triage scoring guidelines for allocation of mechanical ventilators. *JAMA Netw Open*. 2020;3(12):e2029250. doi:10.1001/jamanetworkopen.2020.29250

eFigure 1. Flowchart of Exclusions for Cohort
eFigure 2. Distribution of SOFA Scores Calculated Using the First Available Data in the 24 h After Admission to ICU vs Worst Values in the First 24 h for All Mechanically Ventilated Admissions
eTable 1. List of Exclusion Criteria for Adult Patients for NY State Guidelines
eTable 2. Operational Definitions for Exclusion Criteria for Adult Patients for NY State Guidelines, Using Data Available From Philips eICU Database
eTable 3. Triage Level for Patients Based on Application of Criteria at Time of Admission to ICU Taken From NY State Guidelines
eTable 4. Triage Level for Patients Based on Application of Criteria (A) at 48 h; (B) at 120 h Taken From NY State Guidelines
eTable 5. Number and Percentage of Records Missing Data on Key Variables
eTable 6. Original White & Lo Guidelines; Examples of Major Comorbidities and Severely Life Limiting Comorbidities
eTable 7. Operational Definitions for Major and Severe Life-Limiting Diseases in White & Lo Guidelines, Using Data Available From Philips eICU Database
eTable 8. Point Assignments for Original White & Lo Guidelines
eTable 9. Triage Level for Patients Based on Application of Criteria at Time of Admission to ICU in Original White & Lo Guidelines
eTable 10. Estimates of Percentage of Admissions Who Would Meet Lowest Priority Criteria for Mechanical Ventilation
eTable 11. Characteristics of the Admissions Who Were Lowest Priority in Both Sets of Guidelines vs Other Patients Who Met Criteria for Lowest Priority in Each Guideline
eTable 12. Comparison of Agreement in Classification Regarding Patients in Each Category for Priority for Ventilation on Admission to ICU—Restricted to the First ICU Admission During the Hospitalization
eReferences

This supplementary material has been provided by the authors to give readers additional information about their work.

© 2020 Wunsch H et al. *JAMA Network Open*.

eFigure 1. Flowchart of Exclusions for Cohort

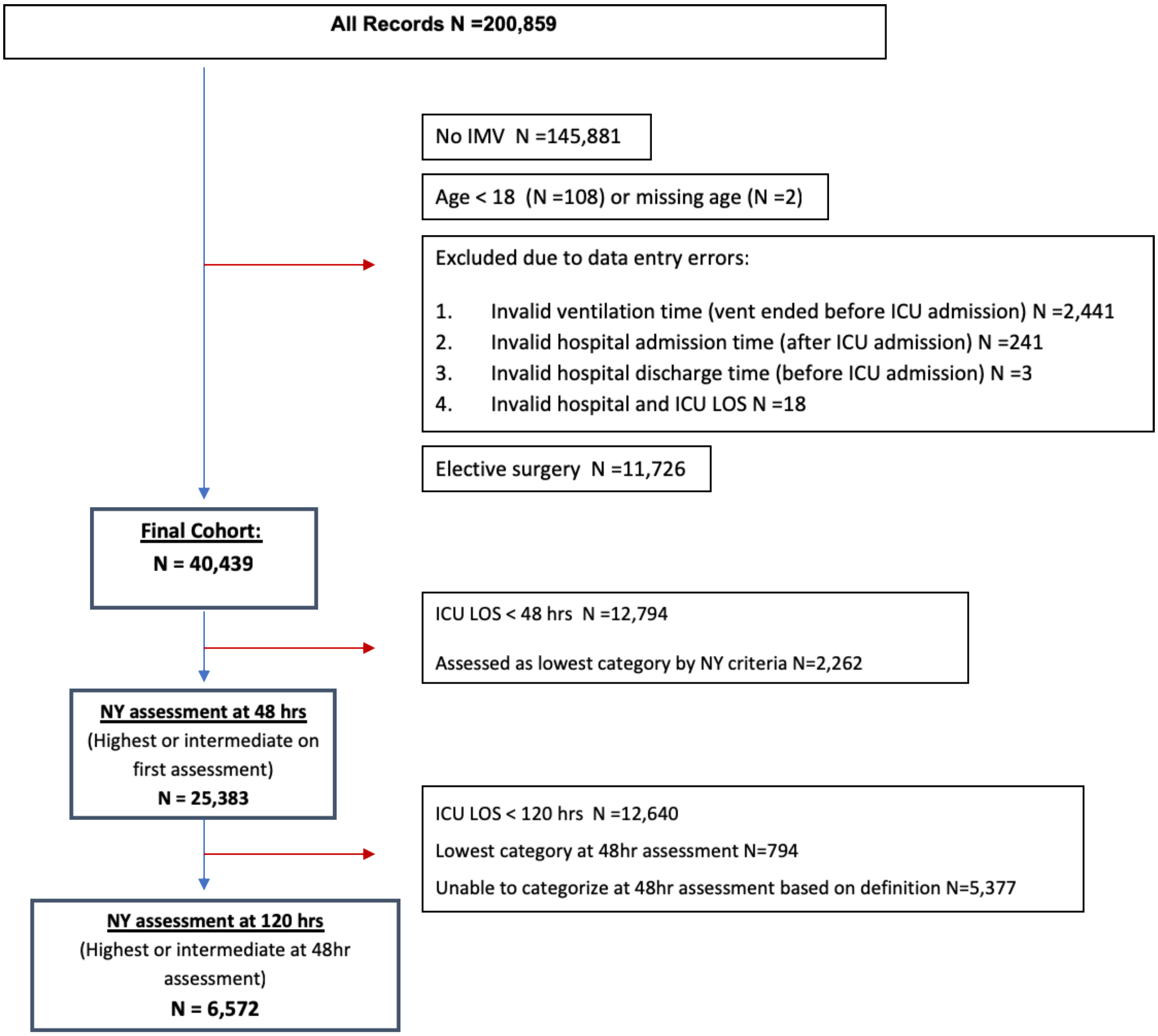

© 2020 Wunsch H et al. *JAMA Network Open*.

eFigure 2. Distribution of SOFA Scores Calculated Using the First Available Data in the 24 h After Admission to ICU vs Worst Values in the First 24 h for All Mechanically Ventilated Admissions

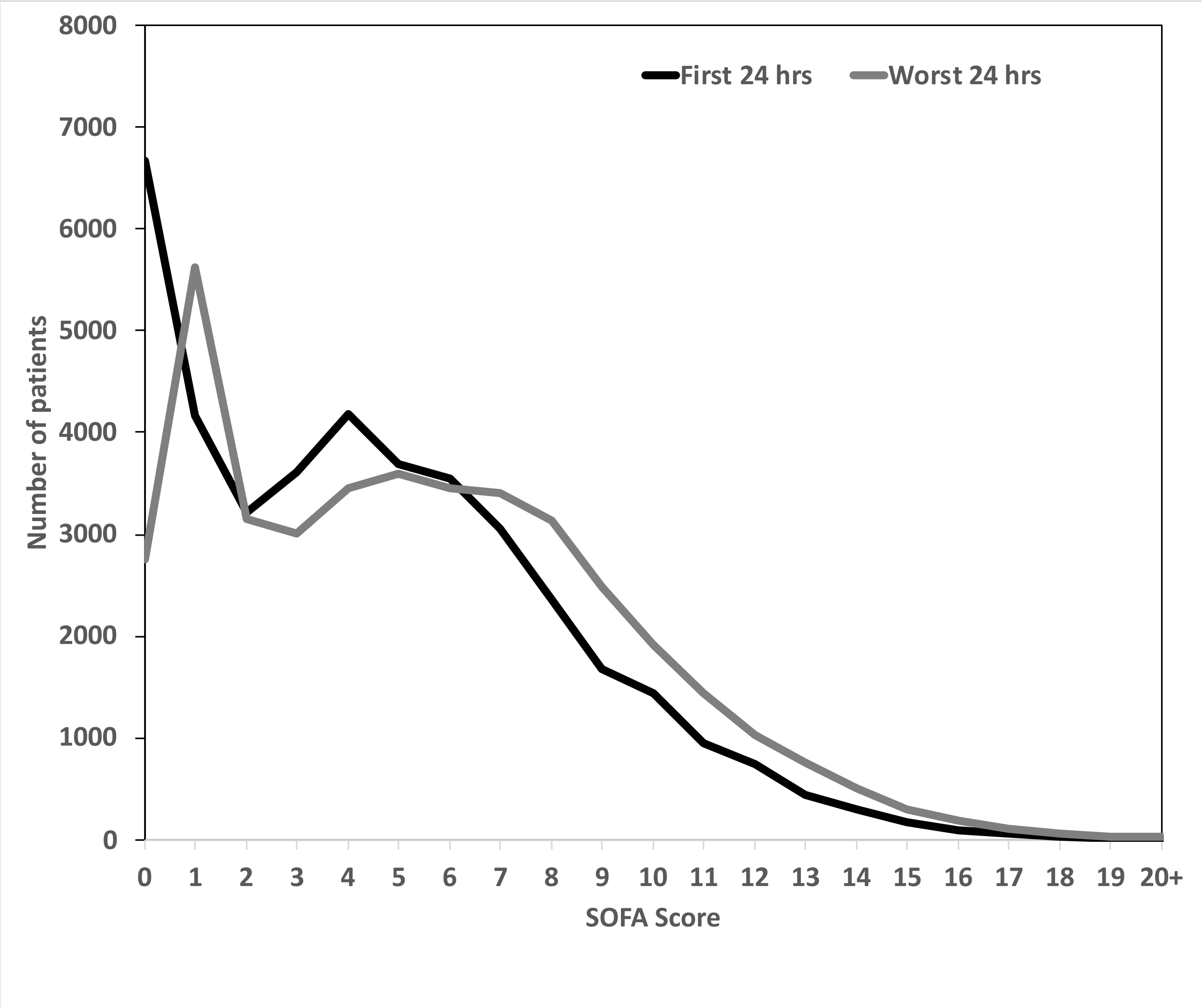

© 2020 Wunsch H et al. *JAMA Network Open*.

eTable 1. List of Exclusion Criteria for Adult Patients for NY State Guidelines[1]

Step 1 - List of Exclusion Criteria for Adult Patients[114]
Medical Conditions that Result in Immediate or Near-Immediate Mortality Even with Aggressive Therapy

- Cardiac arrest: unwitnessed arrest, recurrent arrest without hemodynamic stability, arrest unresponsive to standard interventions and measures; trauma-related arrest
- Irreversible age-specific hypotension unresponsive to fluid resuscitation and vasopressor therapy
- Traumatic brain injury with no motor response to painful stimulus (i.e., best motor response = 1) (See Appendix 1)
- Severe burns: where predicted survival ≤ 10% even with unlimited aggressive therapy (See Appendix 1)
- Any other conditions resulting in immediate or near-immediate mortality even with aggressive therapy[1]

[1] This "catch all" phrase encompasses other possibilities because the list above is merely a guide and does not list every medical condition that would result in immediate or near-immediate mortality.

© 2020 Wunsch H et al. *JAMA Network Open*.

eTable 2. Operational Definitions for Exclusion Criteria for Adult Patients for NY State Guidelines, Using Data Available From Philips eICU Database

Exclusion criteria from NY State Guidelines	Mapping Using Available Philips eICU variables	Additional Notes
**A. Cardiac arrest<br>-unwitnessed arrest<br>-recurrent arrest without hemodynamic stability<br>-arrest unresponsive to standard interventions and measures<br>-trauma related arrest**	APACHE admission diagnosis = cardiac arrest	Estimated percentage of these admission who meet the exclusion criteria using published data [In hospital arrests: 26% unwitnessed, 3.5% trauma; hypotension or hypoperfusion 29.4%].[2]<br>Out of hospital: 58% unwitnessed][3]<br>Estimate top 40% in terms of APACHE IV predicted mortality.
**B. Irreversible age-specific hypotension unresponsive to fluid resuscitation and vasopressor therapy**	Max NE gtt in first 6 hours of admission >50 mcg/min and/or Epi gtt > 50 mcg/min	
**C. Traumatic brain injury with no motor response to painful stimulus (i.e. best motor response = 1)**	APACHE admission diagnosis = Trauma with head component (operative or non-operative) & GCS(motor) = 1	
**D. Severe burns with predicted survival <=10% even with unlimited aggressive therapy**	APACHE admission diagnosis = burn & APACHE IV predicted mortality >90%	This category is subsumed by (E) below
**E. Any other conditions resulting in immediate or near-immediate mortality even with aggressive therapy**	APACHE IV predicted mortality >90% or limitation on life-sustaining therapies on admission to ICU	

© 2020 Wunsch H et al. *JAMA Network Open*.

eTable 3. Triage Level for Patients Based on Application of Criteria at Time of Admission to ICU Taken From NY State Guidelines

Blue = lowest priority; Yellow = intermediate priority; Red = highest priority. Green category was excluded as these individuals did not meet criteria for requiring mechanical ventilation.

Step 1 – Exclusion Criteria: A patient is screened for exclusion criteria, and if s/he has a medical condition on the exclusion criteria list, the patient is not eligible for ventilator therapy. Instead, a patient receives alternative forms of medical intervention and/or palliative care.

Step 2 – Mortality Risk Assessment Using SOFA (Sequential Organ Failure Assessment): A patient is assessed using SOFA, which may be used as a proxy for mortality risk. A triage officer/committee examines clinical data from Steps 1 and 2 and allocates ventilators according to a patient's SOFA score.

Step 3 – Time Trials: Periodic clinical assessments at 48 and 120 hours using SOFA are conducted on a patient who has begun ventilator therapy to evaluate whether s/he continues with the treatment. The decision whether a patient remains on a ventilator is based on his/her SOFA score and the magnitude of change in the SOFA score compared to the results from the previous official clinical assessment.

Step 2 – Mortality Risk Assessment Using SOFA[1]

Color Code and Level of Access	Assessment of Mortality Risk/ Organ Failure
Blue<br>No ventilator provided.<br>Use alternative forms of medical intervention and/or palliative care or discharge.<br>Reassess if ventilators become available.	Exclusion criterion<br>OR<br>SOFA > 11
Red<br>Highest<br>Use ventilators as available	SOFA < 7<br>OR<br>Single organ failure[2]
Yellow<br>Intermediate<br>Use ventilators as available	SOFA 8 – 11
Green<br>Use alternative forms of medical intervention or defer or discharge.<br>Reassess as needed.	No significant organ failure<br>AND/OR<br>No requirement for lifesaving resources

[1] If a patient develops a condition on the exclusion criteria list at any time from the initial assessment to the 48 hour assessment, change color code to blue. Remove the patient from the ventilator and provide alternative forms of medical intervention and/or palliative care.
[2] Intubation for control of the airway (without lung disease) is not considered lung failure.

© 2020 Wunsch H et al. *JAMA Network Open*.

eTable 4. Triage Level for Patients Based on Application of Criteria (A) at 48 h; (B) at 120 h Taken From NY State Guidelines

Blue = lowest priority; Yellow = intermediate priority; Red = highest priority. Green category was excluded as these individuals did not meet criteria for requiring mechanical ventilation.

a. 48 Hour Clinical Assessment Chart

**Step 3 - Ventilator Time Trials (48 Hour Assessment)[1]**	
**Color Code and Level of Access**	**Assessment of Mortality Risk/ Organ Failure**
Blue<br>No ventilator provided.[2]<br>Use alternative forms of medical intervention and/or palliative care or discharge.<br>Reassess if resources become available.	Exclusion criterion<br>OR<br>SOFA > 11<br>OR<br>SOFA 8 – 11 and No Change in SOFA Score Compared to the Initial Assessment[3]
Red<br>Highest<br>Use lifesaving resources as available.	SOFA < 7 and Decrease in SOFA Score Compared to the Initial Assessment[4]<br>OR<br>SOFA < 11 and Decrease in SOFA Score Compared to the Initial Assessment[5]
Yellow<br>Intermediate<br>Use lifesaving resources as available.	SOFA < 7 and No Change in SOFA Score Compared to the Initial Assessment
Green<br>Use alternative forms of medical intervention or defer or discharge.<br>Reassess as needed.	No longer ventilator dependent / Actively weaning from ventilator

[1] If a patient develops a condition on the exclusion criteria list at any time from the initial assessment to the 48 hour assessment, change color code to blue. Remove the patient from the ventilator and provide alternative forms of medical intervention and/or palliative care.
[2] A patient assigned a blue color code is removed from the ventilator and alternative forms of medical intervention and/or palliative care are provided.
[3] The patient remains significantly ill.
[4] These criteria apply to a patient who was placed into the red category at the initial assessment.
[5] These criteria apply to a patient who was placed into the yellow category at the initial assessment but because a ventilator was available the patient began ventilator therapy.

© 2020 Wunsch H et al. *JAMA Network Open*.

b. **120 Hour Clinical Assessment Chart**

**Step 3 - Ventilator Time Trials (120 Hour Assessment)[1]**	
**Color Code and Level of Access**	**Assessment of Mortality Risk/ Organ Failure**
Blue<br>No ventilator provided.[2]<br>Use alternative forms of medical intervention and/or palliative care or discharge.<br>Reassess if resources become available.	Exclusion criterion<br>OR<br>SOFA > 11<br>OR<br>SOFA < 7 and No Change in SOFA Score Compared to the Previous Assessment
Red<br>Highest<br>Use lifesaving resources as available.	SOFA < 7 and Progressive Decrease in SOFA Score Compared to the Previous Assessment
Yellow<br>Intermediate<br>Use lifesaving resources as available.	SOFA < 7 and Minimal Decrease in SOFA Score (< 3 Point Decrease in Previous 72 Hours) Compared to the Previous Assessment
Green<br>Use alternative forms of medical intervention or defer or discharge.<br>Reassess as needed.	No longer ventilator dependent / Actively weaning from ventilator

[1] If a patient develops a condition on the exclusion criteria list at any time from the 48 hour assessment to the 120 hour assessment, change color code to blue. Remove the patient from the ventilator and provide alternative forms of medical intervention and/or palliative care.

[2] A patient assigned a blue color code is removed from the ventilator and alternative forms of medical intervention and/or palliative care are provided.

© 2020 Wunsch H et al. *JAMA Network Open*.

eTable 5. Number and Percentage of Records Missing Data on Key Variables

**Characteristic, No. (%)**	**Total Cohort** (n=40,439)
**Sex**	21 (0.1)
**Race/Ethnicity**	472 (1.2)
**APACHE IV score**	6,033 (14.9)
**APACHE IV predicted mortality**	8,921 (22.1)
**Duration of ventilation**	4,016 (9.9)
**Sequential Organ Failure Assessment (SOFA) components (first 24 hrs)***	
**Respiratory**	13,958 (34.5)
**Central nervous system**	20,047 (49.6)
**Cardiovascular**	1,523 (3.8)
**Liver**	20,323 (50.3)
**Coagulation**	4,633 (11.5)
**Kidney**	3,391 (8.4)
**Care preferences prior to and within 24 hrs of ICU admission**	904 (1.3)
**ICU mortality**	3 (0.01)
**Hospital discharge disposition**	442 (1.1)

Handling of missing data:

For SOFA components: those missing information were assumed to be normal.

For Care Preferences: admissions with missing values were categorized as “Full Therapy”. For patients with more than one care preference specified, the most restrictive preference was used.

© 2020 Wunsch H et al. *JAMA Network Open*.

eTable 6. Original White & Lo Guidelines; Examples of Major Comorbidities and Severely Life Limiting Comorbidities s[4]

Table 2. Examples of Major Comorbidities and Severely Life Limiting Comorbidities*

**Examples of Major comorbidities** (associated with significantly decreased long-term survival)	**Examples of Severely Life Limiting Comorbidities** (commonly associated with survival < 1 year)
• Moderate Alzheimer's disease or related dementia<br>• Malignancy with a < 10 year expected survival<br>• New York Heart Association Class III heart failure<br>• Moderately severe chronic lung disease (e.g., COPD, IPF)<br>• End-stage renal disease in patients < 75<br>• Severe multi-vessel CAD<br>• Cirrhosis with history of decompensation	• Severe Alzheimer's disease or related dementia<br>• Cancer being treated with only palliative interventions (including palliative chemotherapy or radiation)<br>• New York Heart Association Class IV heart failure plus evidence of frailty<br>• Severe chronic lung disease plus evidence of frailty<br>• Cirrhosis with MELD score ≥20, ineligible for transplant<br>• End-stage renal disease in patients older than 75

*This Table only provides examples. There are likely other reasonable approaches to designating 0, 2, or 4 points according to the "save the most life-years" principle. Indices such as Elixhauser or COPS2 may be an option, but these scores may be difficult to calculate quickly.

© 2020 Wunsch H et al. *JAMA Network Open*.

eTable 7. Operational Definitions for Major and Severe Life-Limiting Diseases in White & Lo Guidelines, Using Data Available From Philips eICU Database

Major comorbidities in original White & Lo Guidelines	Mapping Using Available Philips eICU variables	Severely Life Limiting Comorbidities in original White & Lo Guidelines	Mapping Using Available Philips eICU variables
**Moderate Alzheimers or related dementia**	Past medical history "dementia or Alzheimers"	**Severe Alzheimers or related dementia**	Past medical history "dementia or Alzheimers". Then estimated percentage of admissions who meet criteria for severe disease: 45% with cognitive impairment before ICU admission also had severe disability[5]
**Malignancy with <10 year expected survival**	APACHE Past Medical History of metastatic cancer	**Cancer with only palliative interventions**	
**NYHA class III heart failure**	Past medical history "CHF – III"	**NYHA class IV heart failure + frailty**	Past medical history "CHF – IV"
**Mod-severe chronic lung disease**	Past medical history "COPD – moderate"	**Severe chronic lung disease and evidence of frailty**	Past medical history of COPD – severe [note: no information on frailty – considered all patients with severe COPD as frail]
**ESRD patient <75**	Past medical history "end-stage renal disease" & age<75	**ESRD >75**	Past medical history "end-stage renal disease" & age≥75 [included age 75 as not specified by definition]
**Severe multi-vessel CAD**	AMI within 6 months prior; or past medical history of severe CAD		
**Cirrhosis with history of decompensation**	APACHE past medical history of cirrhosis	**Cirrhosis with MELD ≥20, ineligible for transplant**	APACHE Past medical history if cirrhosis + MELD in first 24 hours ≥20)

AMI = acute myocardial infarction; APACHE = acute physiology and chronic health evaluation; CAD = coronary artery disease; CHF = congestive heart failure; COPD = chronic obstructive pulmonary disease; ESRD = end-stage renal disease; MELD = model for end-stage liver disease; PAD = peripheral arterial disease; PVD = peripheral vascular disease

© 2020 Wunsch H et al. *JAMA Network Open*.

eTable 8. Point Assignments for Original White & Lo Guidelines

Principle	Specification	Point System*			
		1	2	3	4
**Save the most lives**	Prognosis for short-term survival (SOFA score#)	SOFA score < 6	SOFA score 6-8	SOFA score 9-11	SOFA score ≥12
**Save the most life-years**	Prognosis for long-term survival (medical assessment of comorbid conditions)	…	Major comorbid conditions with substantial impact on long-term survival	…	Severely life-limiting conditions; death likely within 1 year

© 2020 Wunsch H et al. *JAMA Network Open*.

eTable 9. Triage Level for Patients Based on Application of Criteria at Time of Admission to ICU in Original White & Lo guidelines

Yellow = lowest priority; Orange = intermediate priority; Red = highest priority.
STEP 1: Calculate each patient's priority score using the multi-principle allocation framework.
This allocation framework is based primarily on two considerations: 1) saving the most lives; and 2) saving the most life-years. Patients who are more likely to survive with intensive care are prioritized over patients who are less likely to survive with intensive care. Patients who do not have serious comorbid illness are given priority over those who have illnesses that limit their life expectancy. As summarized in Table 1, the Sequential Organ Failure Assessment (SOFA) score (or an alternate, validated, objective measure of probability of survival to hospital discharge) is used to determine patients' prognoses for hospital survival. In addition, the presence of life-limiting comorbid conditions, as determined by the triage team, is used to characterize patients' longer-term prognosis.

Table 3. Assigning Patients to Color-coded Priority Groups

**Use Raw Score from Multi-principle Scoring System to Assign Priority Category**	
**Level of Priority and Code Color**	**Priority score from Multi-principle Scoring System**
**RED**<br>**Highest priority**	**Priority score 1-3**
**ORANGE**<br>**Intermediate priority**<br>(reassess as needed)	Priority score 4-5
**YELLOW**<br>**Lowest priority**<br>(reassess as needed)	Priority score 6-8

© 2020 Wunsch H et al. *JAMA Network Open*.

eTable 10. Estimates of Percentage of Admissions Who Would Meet Lowest Priority Criteria for Mechanical Ventilation

	Primary estimate, N (%) (SOFA first24)	95% CI	Secondary estimate, N (%) (SOFA worst24)	95% CI	Primary estimate, N (%) (SOFA first24) First admission[2]	95% CI
**NY Priority**						
**Lowest**	3,612 (8.9)	8.7-9.2	4,561 (11.3)	11.0-11.6	3,467 (9.2)	(8.9, 9.5)
**Intermediate**	5,640 (14.0)	13.6-14.3	8,082 (20.0)	19.6-20.4	5,212 (13.8)	(13.5, 14.2)
**Highest[1]**	31,187 (77.1)	76.7-77.5	27,796 (68.7)	68.3-69.2	29,057 (77.0)	(76.6, 77.4)
**White & Lo Priority**						
**Lowest**	1,738 (4.3)	4.1-4.5	2,312 (5.7)	5.5-5.9	1,631 (4.3)	(4.1, 4.5)
**Intermediate**	5,831 (14.4)	14.1-14.8	6,768 (16.7)	16.4-17.1	5,423 (14.4)	(14.0, 14.7)
**Highest**	32,870 (81.3)	80.9-81.7	31,359 (77.5)	77.1-78.0	30,682 (81.3)	(80.9, 81.7)

CI = confidence interval; SOFA = Sequential Organ Failure Assessment

1. Classified as highest priority with a SOFA score of exactly 7: n=3,050
2. First ICU admission during the hospitalization, n=37,736

© 2020 Wunsch H et al. *JAMA Network Open*.

eTable 11. Characteristics of the Admissions Who Were Lowest Priority in Both Sets of Guidelines vs Other Patients Who Met Criteria for Lowest Priority in Each Guideline

	Lowest Criteria		
Characteristic	Both Guidelines	NY State only	White & Lo only
Number of patients, n (%) [95% CI]	655 (1.6) [1.5-1.7]	2,957 (7.3) [7.1-7.6]	1,083 (2.7) [2.5-2.8]
Percentage of total ventilation hours, % [95% CI]	2.0 [1.9-2.0]	8.5 [8.4-8.6]	3.1 [3.0-3.1]
Age years mean (SD)	64.6 (14.1)	62.5 (17)	71.0 (12.0)
Male sex, No. (%)	382 (58.4)	1,766 (59.9)	582 (53.7)
Race/Ethnicity, No. (%)			
White	451 (69.2)	2,167 (74.1)	793 (73.6)
Black	100 (15.3)	344 (11.8)	142 (13.2)
Asian	4 (0.6)	44 (1.5)	9 (0.8)
Hispanic	39 (6)	148 (5.1)	71 (6.6)
Other/Unknown	58 (8.9)	222 (7.6)	62 (5.8)
ICU type, No. (%)			
Medical	61 (9.3)	296 (10)	130 (12)
Medical/Surgical	420 (64.1)	1,622 (54.9)	686 (63.3)
Surgical	29 (4.4)	189 (6.4)	37 (3.4)
Neurological	16 (2.4)	136 (4.6)	28 (2.6)
Cardiac/Cardiothoracic	129 (19.7)	714 (24.1)	202 (18.7)
APACHE IV score, mean (SD)	122 (30.4)	120.1 (26.6)	88.6 (25.6)
APACHE IV predicted mortality, mean (SD)	69.5 (25.4)	71.6 (22.3)	41.0 (23.1)
Sequential Organ Failure Assessment score, mean (SD)[2]	12.8 (2.4)	9.5 (4.3)	7.7 (1.5)
Duration of ventilation (hrs), median (IQR)	61.7 (22.8, 148.5)	56.8 (19.5, 131.3)	61.8 (24.9, 138.8)
Care preferences prior to and within 24 hrs of ICU admission, N (%)			
Full therapy	492 (75.1)	2,320 (78.5)	849 (78.4)
Do not resuscitate	104 (15.9)	441 (14.9)	149 (13.8)
Limitations on life support/other care	59 (9)	196 (6.6)	85 (7.8)

© 2020 Wunsch H et al. *JAMA Network Open*.

**ICU length of stay, days (IQR)**	3.3 (1.4, 7.3)	3.1 (1.1, 6.9)	3.7 (2, 7)

© 2020 Wunsch H et al. *JAMA Network Open*.

eTable 12. Comparison of Agreement in Classification Regarding Patients in Each Category for Priority for Ventilation on Admission to ICU—Restricted to the First ICU Admission During the Hospitalization

Percentages Refer to the Entire Cohort

N=37,736	**White & Lo Priority Score**		
**New York Priority Score**	**6-8 (lowest priority)**	**4-5 (intermediate priority)**	**1-3 (highest priority)**
**Blue (lowest priority)**	**613 (1.6)**	1,433 (3.8)	1,421 (3.8)
**Yellow (intermediate priority)**	475 (1.3)	**977 (2.6)**	3,760 (10.0)
**Red (highest priority)**	543 (1.4)	3,013 (8.0)	**25,501 (67.6)**

Kappa for agreement assessing lowest priority versus other categories combined (intermediate priority and highest priority)=0.19 (95% CI 0.18-0.21)

© 2020 Wunsch H et al. *JAMA Network Open*.

eReferences

1. *Ventilator Allocation Guidelines: New York State Task Force on Life and the Law* New York State Department of Health;2015.
2. Goldberger ZD, Chan PS, Berg RA, et al. Duration of resuscitation efforts and survival after in-hospital cardiac arrest: an observational study. *Lancet.* 2012;380(9852):1473-1481.
3. Nichol G, Thomas E, Callaway CW, et al. Regional variation in out-of-hospital cardiac arrest incidence and outcome. *JAMA.* 2008;300(12):1423-1431.
4. White DB, Lo B. A Framework for Rationing Ventilators and Critical Care Beds During the COVID 19 Pandemic. *JAMA.* 2020;323(18):1773-1774.
5. Ferrante LE, Pisani MA, Murphy TE, Gahbauer EA, Leo-Summers LS, Gill TM. Functional trajectories among older persons before and after critical illness. *JAMA internal medicine.* 2015;175(4):523-529.

© 2020 Wunsch H et al. *JAMA Network Open*.
